# Comparing the remineralization potential of undemineralized dentin powder versus chicken eggshell powder on artificially induced initial enamel carious lesions: an in-vitro investigation

**DOI:** 10.1186/s12903-024-04778-6

**Published:** 2024-09-08

**Authors:** Mai Badreldin Helal, Mai Samy Sheta, Wafaa Yahia Alghonemy

**Affiliations:** 1 Oral Biology Department, Faculty of Dentistry, Tanta University, El-Giesh St, Tanta, Gharbia Egypt; 2 Dental Biomaterial Department, Faculty of Dentistry, Tanta University, El-Giesh St, Tanta, Gharbia Egypt; 3 Department of Basic Medical and Dental Sciences, Faculty of Dentistry, Zarqa University, Zarqa City, Jordan; 4 Oral Biology Department, Faculty of Dentistry, Tanta University, El-Giesh St, Tanta, Gharbia Egypt

**Keywords:** Chicken eggshell powder, Demineralization, Undemineralized dentin matrix, Enamel remineralization

## Abstract

**Background:**

White spot lesions are a widespread undesirable effect, especially *prevalent during fixed orthodontic treatments*. The study compared the in vitro enamel remineralization potential of undemineralized dentin matrix (UDD) versus chicken eggshell powder (CESP) for artificially induced enamel lesions.

**Methods:**

100 caries-free and sound maxillary premolars were randomly divided into four groups each contain 25 teeth: Group I (Baseline): No treatment was done to the enamel surface. Group II (Negative control ): The enamel surface of the teeth underwent demineralization using demineralizing solution to create artificial carious lesions then kept in artificial saliva. Group III (CESP treated): After demineralizing the tooth surface, the teeth have been suspended in the CESP remineralizing solution. Group IV (UDD treated): After enamel demineralization, the teeth were suspended in UDD remineralizing solution. The remineralization potential was assessed by Vickers microhardness testing, scanning electron microscopic examination (SEM), and energy dispersive X-ray (EDX).

**Results:**

The current study demonstrated an increase in the mean microhardness of CESP and UDD-treated groups; however, It was nearer to the baseline level in the UDD group. SEM imaging revealed greater enamel remineralization in the UDD group compared to the remaining groups. The UDD group disclosed complete coverage for the prismatic enamel compared to the CESP group, which revealed a partially remineralized enamel surface. Interestingly, the Ca/P ratio increased significantly in the CESP group compared to the negative control group. In contrast, a higher significant increase in the mean Ca/P ratios was recorded in the UDD group compared to the test groups.

**Conclusion:**

biomimetic UDD and CESP powder should be utilized to treat enamel early carious lesions. However, UDD demonstrated the most significant remineralization potential.

**Supplementary Information:**

The online version contains supplementary material available at 10.1186/s12903-024-04778-6.

## Introduction

Enamel is a valuable, highly mineralized, epithelial-derived tissue in the body. Due to enamel’s unique characteristics, several studies have investigated the initial demineralization of enamel or white spot lesions [1]. These white spot lesions are frequently related to molar-incisor hypomineralization [2] and dental trauma [3]. Also, it is commonly developed among patients receiving orthodontic treatment [4, 5]. On the molecular level, white spot lesion starts with subsurface dissolution of the enamel crystals, creating micropores within the enamel structure. The latter manifests as white spots that persist and cause aesthetic problems [6].

Interestingly, it has been reported that good oral hygiene and topical fluoride application might minimize enamel demineralization [7]. The latter treatment approach depends on fluoride ions deposition and integration within the tooth minerals forming fluorohydroxyapatite crystals, thus strengthening the tooth structure (7). Also, many researchers have demonstrated the efficacy of chicken eggshell powder (CESP) as a naturally occurring bioactive substance in preserving enamel quality and preventing carious lesions [8–11]. CESP was proven to have a remineralization potential for the initial artificially induced enamel carious lesions [12, 13] as CESP is considered an enriched bioavailable calcium source that may contribute to reducing enamel demineralization ability [14]. Additionally, the CESP was used to treat various human health problems, such as osteoporosis, when administered orally for a year [15]. It also was portrayed to increase bone mineral density in experimentally induced osteoporosis after ovariectomy in female rats [16].

Undemineralized dentin matrix (UDD) and demineralized dentin matrix (DDM) have recently been considered exclusive natural bioactive byproducts of dentin. DDM is a biocompatible and osteoinductive material [17]. Furthermore, DDM was found to induce bone regeneration in critical-size bony defects, treating teeth sockets after extraction [18], and periodontal regeneration [19]. Interestingly, UDD was proven to have high crystalline content, allowing it to be used in remineralization.

To our knowledge, the efficiency of the biomimetic remineralizing agents in treating the initial enamel demineralization is still uncertain. Also, no prior study used UDD to treat white spot lesions. Therefore, this in vitro study aimed to compare the enamel remineralization ability of UDD versus CESP for artificially induced enamel carious lesions. The scientific null-hypothesis asserted that undemineralized dentin powder do not differ significantly in the enamel remineralization ability compared to chicken eggshell powder.

## Materials and methods

A total of 100 maxillary premolars extracted for orthodontic purposes were used in this study. The Epi-Info statistical software tool was used to calculate the sample size. The sample size was calculated using the following criteria: 95% confidence level and 88% power. The teeth were selected based on specified inclusion and exclusion criteria. Our inclusion criteria included removed, sound, non-carious, and unrestored first premolars. Caries, periodontitis, restoration, visible cracks, or anomalies were all considered exclusion criteria. Informed patient consent was obtained by the rules for human research subjects established by the Ethical Review Board at the Faculty of Dentistry in Egypt (ethical approval #R-OB-9-22-1).

### Sample preparation and grouping

All calculus and soft debris were removed from teeth by a hand scaler and washed thoroughly with distilled water. The external enamel surfaces of all teeth were polished using rotary discs and silicon carbide abrasive papers. The teeth were decornated at CEJ by a water-cooled low-speed saw (Isomet^®^ 5000 Linear Precision Saw, Buehler Ltd, Bluff, IL). Then, the coronal portions of each tooth were embedded into blocks of self cure acrylic resin, while the buccal surfaces facing upward. Pieces of modeling wax sheet measuring 4 × 4 mm were cut and inserted in the middle third of the buccal surfaces of the teeth. On the remaining parts of the teeth, an acid-resistant varnish was applied and allowed to dry. The wax sheet was then removed to create a 4 × 4 mm window on the enamel surface, in which the lesion was created. Specimens were kept in artificial saliva until subsequent use.

The prepared specimens were randomly divided into one of four groups based on a computer-generated list of random numbers as follows:

- **Group I (Baseline)** (*n* = 25): The teeth were stored in artificial saliva without any applied treatment to the enamel surface.
- **Group II (Negative control group) (-ve CTL)** (*n* = 25): The teeth underwent enamel surface demineralization and were then kept in artificial saliva for seven days.
- **Group III (CESP group)** (*n* = 25): Following demineralizing the tooth surface, the samples were suspended in CESP remineralizing solution twice daily for seven consecutive days.
- **Group IV (UDD group)** (*n* = 25): The teeth underwent enamel surface demineralization. Then, the UDD solution was used as a remineralizing agent twice daily for seven days.

**Fig. 1:**
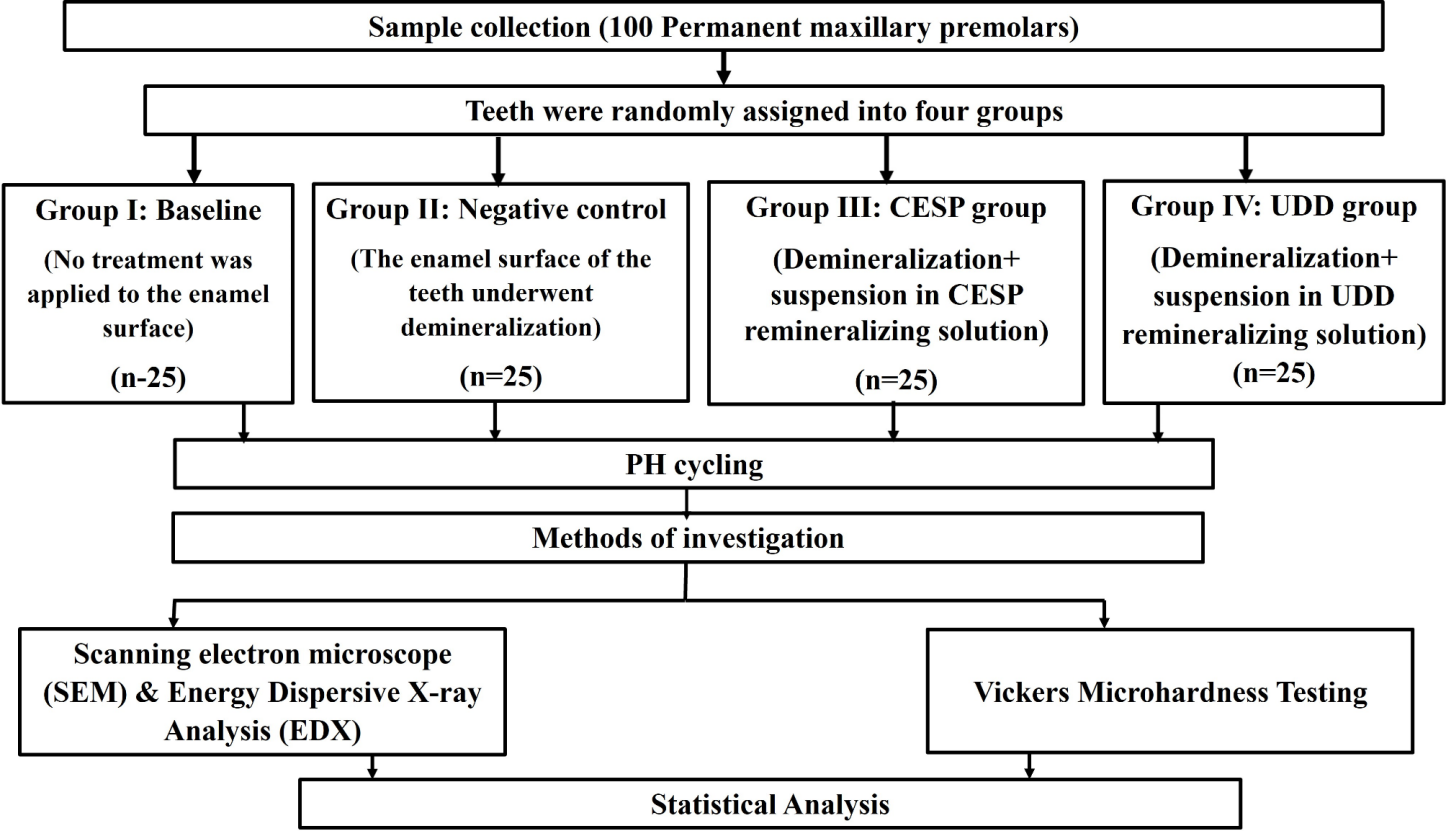
Flow chart explaining the study design

Afterward, the specimens were subjected to PH cycling five days after the experimental procedure. The specimens were evaluated for microhardness testing, energy dispersive X-ray spectroscopy (EDX), and SEM. These testing steps were done for all specimens at baseline (before any surface treatment), after the demineralization protocol, after applying the natural remineralizing agent, and after the pH cycle. The study design is shown in Fig. 1.

### Experimental procedure

The current study utilized two remineralizing agents: laboratory-prepared CESP and UDD. The study materials are shown in Table 1.

**Table 1:** Table illustrating the study materials preparation and composition

Materials	preparation	composition
**Chicken egg shell powder**	Laboratory prepared	66% calcium carbonate,1% calcium phosphate, 1% magnesium carbonate.
**Undemineralized dentin powder**	Laboratory prepared	90% calcium carbonate,5% calcium phosphate, 6% magnesium carbonate
**Artificial saliva**	Laboratory prepared	(Na-3PO4 (3.90mM), NaCl (4.28mM), KCI (17.99mM), CaCl2 (1.11mM), MgCl2 (0.09mM), H2SO4 (0.50mM), NaHCO3 (3.28 mM) [22].
**Demineralizing solution**	Laboratory prepared	2.2 m Mol calcium chloride, 0.05 M acetic acid, 2.2 mM sodium phosphate, and 1 M potassium hydroxide with PH 4.5. [20, 21].
**PH cycling solution**	Laboratory prepared	1.5 m Mol calcium chloride, 0.9 m Mol sodium phosphate, and 0.15 m Mol potassium chloride with pH 7.0. The demineralizing solutions were composed of 2.2 m Mol calcium chloride, 0.05 M acetic acid, 2.2 mM sodium phosphate, and 1 M potassium hydroxide with PH 5.0. [21].

**Table 2:** Means, standard deviation values of Vickers microhardness in all tested group

	Group I Baseline	Group II Negative control	Group III CESP	Group IV UDD
Baseline	375 ± 9.27^aA^	373.50 ± 8.66^aA^	374.50 ± 10.85^aA^	375.25 ± 10.34^aA^
Demineralization	-----------	270.25 ± 11.96^aB^	267.25 ± 13.53^aB^	268.50 ± 14.34^aB^
Remineralization	-----------	293.75 ± 7.89^aC^	317.50 ± 10.72^bC^	349 ± 16.10^cC^

Different lowercase letters indicate statistically significant difference in rows. Different uppercase letters indicate statistically significant difference in columns (*p* < 0.05)

**Table 3:** Means, standard deviation values of the (Ca/P) ratio obtained by EDX analysis in all tested groups:

	Group I Baseline	Group II Negative control	Group III CESP	Group IV UDD
Baseline	1.04 ± 0.49^aA^	0.80 ± 0.24^aA^	0.88 ± 0.36^aA^	1.04 ± 0.49^aA^
Demineralization	-----------	0.54 ± 0.21^aB^	0.46 ± 0.27^aB^	0.46 ± 0.24^aB^
Remineralization	------	0.54 ± 0.23^aB^	1.08 ± 0.33^bA^	1.94 ± 0.11^cC^

Different lowercase letters indicate statistically significant difference in rows. Different uppercase letters indicate statistically significant difference in columns (*p* < 0.05)

## Demineralization step (White spot lesion creation)

Specimens from groups II, III, and IV were demineralized by immersion in 30 ml of demineralizing solution for 96 h to create artificial carious lesions on the exposed enamel surface. The demineralizing solution was prepared according to Yadav et al. [20] protocol by adding (2.2 m Mol calcium chloride, 0.05 M acetic acid, 2.2 mM sodium phosphate, and 1 M potassium hydroxide) The final pH was adjusted to 4.5 with 50% sodium hydrodroxide. The solution was changed every 24 h to maintain solution effectiveness. The demineralizing solution composition [20, 21] is shown in Table 1.

## Remineralization step

- **Undemineralized Dentin Matrix (UDD) application**.

**Fig. 2:**
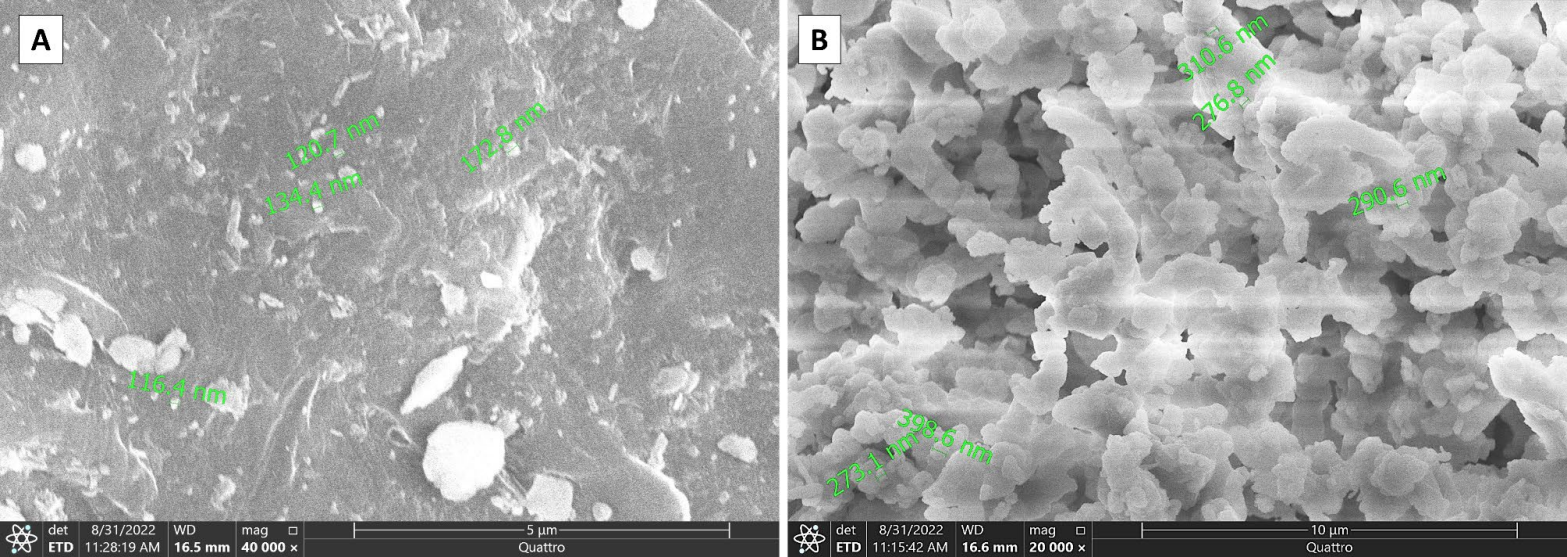
Scanning electron micrographs showing: **(A)** The particle size of UDD. **(B)** The particle size of calcinated CESP

Ten extracted human teeth were taken to prepare fine UDD. The procedure used for UDD production was similar to that of Haapasalo et al. [17]. The UDD was obtained from the extracted teeth after the removal of any calculus, residues of the pulp, and periodontal ligament. A low-speed motor removed enamel and cementum, and dentin particles were produced by crushing the remaining dentin in the milling machine. SEM and elemental analysis were used to determine the dentin particle size and composition (Fig. 2-A, Table 1). A 3% UDD suspension was prepared by dissolving one gram of the powder in 33.3 ml of sterile distilled water. The demineralized specimens in group IV were immersed in the 3% UDD suspension twice daily with a 12-hour interval between each immersion. This cycle was repeated using a freshly prepared solution for seven days. Specimens were removed from the solution, rinsed in distilled water, and stored in artificial saliva [22] until subsequent use.

b. **Eggshell powder solution application**.

CESP was prepared and characterised according to the protocol given by Tangboriboon et al. [23]. Characterization and composition of CESP particle size were done by SEM and elemental analysis, respectively (Fig. 2-B, Table 1). Subsequently, a 3% CESP solution was then produced by dissolving one gram of the powder in 33.3 ml of sterile distilled water. Then, the demineralized specimens in the CESP group were immersed in the freshly prepared solution for seven days. The specimen immersion was done twice daily with a 12-hour delay between each immersion. Specimens were removed from the solution, rinsed in distilled water, and kept in artificial saliva until use.

### PH cycling

Over 5 days, all group specimens were subjected to remineralization/demineralization pH cycles. Specimens were successively subjected to 30 ml of demineralization solution for 3 h, then immersed in 30 ml of remineralizing solution for 21 h. The composition of the remineralizing solutions and the demineralizing solutions [21] are shown in Table 1.

### Microhardness testing

Ten specimens from each group were subjected to surface microhardness testing by a Vickers microhardness testing machine (Zwick/Roell, INDENTEC, ZHVµ-S, West Millands, England). Specmins microhardness was evaluated throughout the three study stages before demineralization, after demineralization and after remineralization. A load of 300 g was applied for a 20 s dwell time, and each specimen received three indentations separated by 100 microns. The mean hardness value for each sample was calculated by averaging the value of all three indentations.

### Scanning electron microscopic examination (SEM) and Energy dispersive X-ray (EDX) of the specimens

Throughout the phases of the study, testing was performed. Fifteen specimens from each group were subjected to a quantitative evaluation of their calcium and phosphate content on the enamel surface. Testing was done using SEM equipped with energy-dispersive X-ray spectroscopy [EDX]. Then, all specimens in each group were also examined under SEM to assess enamel structure.

For SEM analysis, **s**amples underwent fixation in a solution comprising 2.5% glutaraldehyde and 2% paraformaldehyde dissolved in 0.1 M phosphate buffer (pH 7.2–7.4) for a duration extending from 24 to 48 h at a temperature of 4 °C. Specmins were then supjected to distilled water rinse and immersion in a 0.5% sodium hypochlorite (NaOCl) solution for a period of one hour at ambient temperature. Following this step, a twelve hour rinse was done at temperature of 4 °C before being post-fixed in 1% osmium tetroxide for 2 h at 4 °C. The next stage involved the dehydration process using asscending concentrations of ethanol (70%, 80%, 90%, 95%, and 100%) and desiccation with liquid CO2 in a critical point dryer. Lastly, the specimens were pinned to metal stubs that had been coated with gold/palladium through ion sputtering.

### Statistical analysis

The quantitative data from EDX and Vickers hardness analyses were collected, tabulated, and statistically analyzed using CO-STAT analysis (version 6.4). Descriptive statistics expressed numerical variables as mean, standard deviation, and range. The one-way ANOVA test and the post-hoc test (Tukey test) were used to compare quantitative data between groups.

## Results

### Statistical analysis of Vickers hardness analysis

The means and standard deviation (SD) of Vickers microhardness for all specimens are shown in (Table 2). It disclosed a significant increase in surface microhardness of CESP and UDD-treated specimens when compared with its demineralized values. Multiple comparisons Tuckey tests showed that there was a substantial increase in surface microhardness of the CESP group (*p* < 0.05) and a highly significant increase (*p* < 0.001) recorded by the UDD group when compared to the -ve CTL group. The UDD group showed an increase in the mean surface microhardness of enamel closer to the baseline values.

### Scanning electron microscopic results

**Fig. 3:**
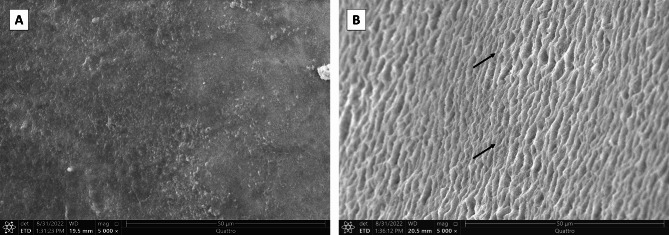
Scanning electron micrograph of enamel surface: **(A)** (Baseline group) showing a smooth layer of a prismatic surface layer of enamel. **(B)** (-ve CTL group) showing demineralized enamel surface with obvious dissolution of the rod substance (black arrows)

**Fig. 4:**
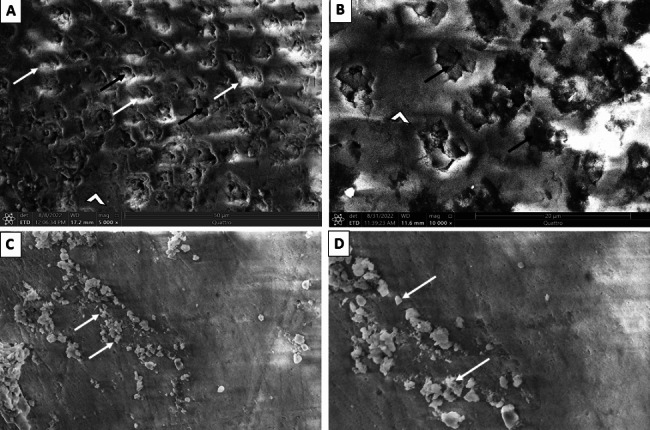
Scanning electron micrograph of enamel surface: **(A)** CESP group showing partially remineralized enamel surface. Notice, some areas exhibit remineralized homogenous enamel (white arrowhead), whereas others reveal areas of demineralized enamel rods (black arrow). **(B)** Higher magnification of enamel surface in CESP group displaying remineralized smooth enamel surface (arrowhead) together with some sort of demineralized enamel rods (black arrows). **(C)** UDD group illustrating completely remineralized enamel surface together with areas of calcified deposits (white arrows) on the enamel surface. **(D)** Higher magnification of enamel surface in UDD group illustrating completely remineralized enamel surface with newly deposited crystallites (white arrows)

**Fig. 5:**
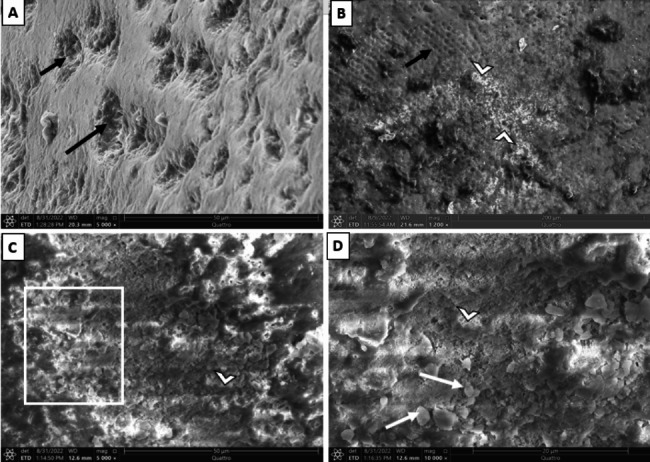
Scanning electron micrograph of enamel surface: **A.** (-ve CTL group) displaying depressions resulted from dissolution of the bodies of enamel rods (black arrows). **B.**CESP group showing remnants of CESP (arrow heads) on the partially remineralized enamel surface. Notice, some areas exhibit demineralized enamel rods (black arrow). **C.** (UDD group) illustrating completely remineralized enamel surface (arrowhead) with crystallites deposition (squared area) on the enamel surface. **D.** Higher magnification of the squared area illustrating completely remineralized enamel surface (arrowhead) with numerous coalesced crystals (white arrows)

Ultrastructurally, untreated enamel surface in baseline group appeared uniform with a homogenous, occasionally smooth aprismatic surface layer of enamel (Fig. 3- A). Conversely, the enamel surface layer of the -ve CTL group demonstrated partial dissolution of the aprismatic enamel and noticeable dissolution of the enamel rods’ bodies (Fig. 3- B). Noticeably, the CESP group demonstrated a partially remineralized enamel surface. Some areas exhibit remineralized homogenous enamel, whereas others still reveal areas of demineralized enamel rods (Figs. 4- A and B and 5- B). Some teeth exhibited a prismatic surface layer with depressions of variable size (Fig. 5- A). Interestingly, the UDD group exhibited greater remineralization for the enamel surface with complete coverage for the colorful enamel. Moreover, the enamel surface in the UDD group appeared covered with newly deposited coalesced globular calcified crystallites (Figs. 4- C and D and 5- C and D).

### Statistical analysis of the (ca), (P) content, and (Ca/P) ratio obtained by EDX analysis

Calcium and phosphorus weight% of the specimens were measured using EDX spectrometry. Then, the calcium and phosphorus content was converted into Ca/P ratios for each group. Regarding the Ca/P ratio, the mean and standard deviation (SD) of all specimens are shown in (Table 3). Statistical analysis using the post-hoc test (Tukey test) revealed that there was no significant increase in the Ca/P ratio in Group II after remineralization when compared with its demineralized values. In contrast, a substantial increase in the Ca/P ratio was reported in Group III after treatment (*p* < 0.05), which was not significantly different from the baseline. Interestingly, Group IV demonstrated a higher significant increase in the mean Ca/P ratios (*p* < 0.001) than the test groups after remineralization, with their values also significantly higher than the baseline.

## Discussion

The current research emphasized the role of UDD versus CESP in enamel remineralization after artificially induced enamel carious lesions. The null hypothesis was rejected in this study as our microhardness testing and SEM imaging results indicated that UDD remineralizing capacity was higher than CESP.

In our study, artificial caries on enamel were applied to individual teeth by immersing them in a demineralizing solution [21] to induce a cariogenic challenge on the facial surface. This standard is comparable to the clinical condition of white lesions developed with fixed orthodontic appliances. The teeth underwent the **PH cycling procedure** to ensure the acid resistance ability of the specimens, remove pathogens, and increase their alkalinity [24]. Regarding the procedures used to asses enamel remineralization, Microhardness measurement is considered a non-destructive, simple, and quick method for measuring non-homogenous materials with microstructure and susceptible to surface cracking, such as enamel [25]. In addition, **SEM analysis** of the tooth surface in all groups was employed to evaluate the samples’ surface morphology, spot the emergence of hydroxyapatite crystals, and verify the materials’ bioactivity. as remineralizing agents [26].

**White lesions** or caries are formed as an initial form of caries that occurs on the enamel surface of dental plaque accumulation areas such as around orthodontic brackets and underneath bands of fixed orthodontic appliances [27] because it’s challenging to practice traditional oral hygiene on teeth surfaces where dental caries isn’t joint [28]. Therefore, numerous clinical surveys have demonstrated a higher incidence of carious lesions on the facial and lingual surfaces of the tooth crown during orthodontic treatment [6, 29]. The initial dental caries (white spots) usually include unbalanced demineralization and remineralization of the enamel due to the dissolution caused by organic acids formed by the dental plaque bacteria [30]. Significantly, demineralization results from the loss of calcium and phosphate ions. These initial defects can be repaired using a non-invasive calcium phosphate protocol [31, 32].

Interestingly, CESP has been identified as a promising biomimetic substance, Owing to its well-documented efficacy in promoting enamel remineralization together with its wide range of application in several medical and dental fields [15–17, 32]. *For instance*,* the unique role of CESP in enhancing remineralization process has been demonstrated. This was confirmed through previous studies* showing that CESP-driven nanohydroxyapatite can effectively seal dentinal tubules and their lateral extensions in treated root canals when combined with sodium *fluoride* [33]. *Furthermore*, the combined application of CESP and nanohydroxyapatite crystals on bleached enamel specimens has resulted in the highest microhardness and the lowest surface roughness compared to untreated samples [34].

Regarding **Vickers microhardness measurements**, The microhardness of enamel in group III (CESP group) was significantly higher than that of demineralized enamel. This finding could be attributed to the well-known CESP nature as an enriched source of Ca [35]. According to the fluorescence spectroscopic analysis of CESP, it consists of approximately 0.53% magnesium, 0.18% strontium, 0.46% phosphate, 0.03% potassium, and 98% calcium [14]. When CESP was administered topically, this high Ca concentration was essential for enamel remineralization [14]. The pH of the CESP solution, as determined by a pH meter, was reported to be 11.2. This increased pH encourages the ionic activity of anions, such as hydroxyl and phosphate ions, to increase. As a result, more ions are accessible for remineralizing the enamel surface. These findings are consistent with those reported by Mony et al. [14]. They concluded that CESP, with its high pH and abundant bioavailable calcium concentration, has the potential to encourage remineralization. These findings also align with Hadidi et al. [36], who reported that incipient enamel carious lesions could benefit from the remineralizing effects of eggshell solution., and it was proved to be an efficient nano-hydroxyapatite suitable for enamel remineralization. Moreover, Yaberi and Haghgoo [37] reported that enamel microhardness significantly increased after eggshell extract solution were applied. Calcium and phosphate ion accessibility are essential for remineralization, and the solution’s elevated pH and high bioavailability of phosphate and calcium ions are primarily responsible for the remineralization process [38]. Therefore, remineralization is facilitated, and tooth surface demineralization is prevented by maintaining a high concentration of calcium and phosphate ions [39]. Due to the high bioavailability of phosphate and calcium ions and an increase in pH, CESP application results in remineralization.

Regarding group IV (UDD treated specimens), u*ndemineralized dentin is considered as anovel biomemic material used in our investigation. UDD powder has not been specifically mentioned in the provided contexts for enamel remineralization. Though*,* El Kady et al.* [40] *compared the efficacy of demineralized (DDM) and undemineralized dentin matrix (UDD) versus hybrid dentin matrices (UDDM + UDD) on mandibular bone defects regeneration in rabbits. They found that both DDM and UDD induced bone regeneration*,* with superior regeneration ability of the hyprid matrix. Additionally*,* researchers portrayed that DDM can promote osteogenic differentiation of stem cells*,* which is important for bone regeneration. The distinct ability of DDM to promote bone healing was attributed to its capacity to form collagen-based osteoinductive scaffolds that aids in tissue engineering*,* facilitated by its organic matrix components collagen type I and non-collagenous proteins and hydroxyapatite crystals* [41]. *These matrix components could potentially provide UDD with superior abilitities to pormote enamel remineralization*,* via inducing the perciptation of hyproxyappatite crystals* [42]. *The later was more confirmed in our expermint*,* as* a higher levels of microhardness and Ca/p ratio was observed in UDD treated samples compared to those treated with CESP.

**Scanning electron microscopic results** of group I (baseline) revealed a homogenous, smooth, colorful surface layer of enamel. On the contrary, the enamel surface layer of group II (-ve CTL) revealed evident dissolution of the bodies of enamel rods because of the demineralizing solutions. Group III (CESP) demonstrated a partially remineralized enamel surface. Some areas exhibit remineralized homogenous enamel, whereas others still reveal areas of demineralized enamel rods. This finding could be attributed to the remineralization of initially demineralized enamel using CESP and a positive effect on both calcium and phosphorus levels after exposure of the specimens to CESP solution, as shown by a previous study [43]. Interestingly, the UDD group divulged greater remineralization for the enamel surface with complete coverage for the colorful enamel. Moreover, the enamel surface in Group IV seemed to be covered with newly deposited coalesced globular calcified crystallites. This finding aligns with Lata et al. [25], who found that in vitro remineralization of demineralized enamel involves several stages, including the formation and growth of new crystals and the regrowth of preexisting crystals. A higher ratio of phosphate and calcium in the UDD powder solution diffuses into the tooth, and utilizing fluoride (in artificial saliva) improves the crystals in terms of purity. i.e., the formation of fluoridated apatite with less carbonate and magnesium, as described by Grohe et al. [44]. Based on the current study findings, UDD and CESP powder reduced enamel demineralization ability and increased enamel remineralization potential, with the UDD powder group reporting the most significant remineralization results.

To our knowledge, this is the first study to document UDD powder’s better capacity as a remineralizing agent in treating the artificially induced initial enamel carious lesions. Thus, bioactive UDD should be used by the dentists to improve surface hardness and restore the surface morphological changes induced by demineralization.

### Study limitation

This research lacked commercial gold standard product such as products containing sodium fluoride and calcium phosphate, given that its primary aim was to evaluate the remineralization potential of two biomemic materials UDD versus CESP after artificial induction of initial enamel carious lesion. In other words, this study focused on the treatment of initial caries by biomimetic material to accept or reject the null hypothesis for UDD and CESP.

## Electronic supplementary material

Below is the link to the electronic supplementary material.

Supplementary Material 1

## Data Availability

Availability of data and materials: All data of this study are available from the corresponding author upon request.
