# Supplementary figures and images for: Comparing the remineralization potential of undemineralized dentin powder versus chicken eggshell powder on artificially induced initial enamel carious lesions: an in-vitro investigation
Source: BMC Oral Health. 2024 Sep 8;24:1048. doi: 10.1186/s12903-024-04778-6 (PMC11382454; doi:10.1186/s12903-024-04778-6)

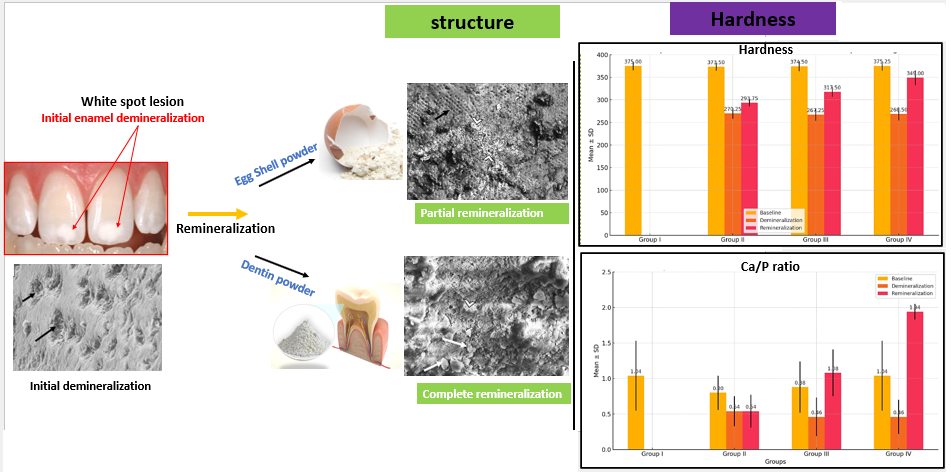

Supplement: Supplementary file 1 — Supplementary Material 1 [file 12903_2024_4778_MOESM1_ESM.tif]
